# CALR mutation characterization in myeloproliferative neoplasms

**DOI:** 10.18632/oncotarget.10376

**Published:** 2016-07-01

**Authors:** Cristina Bilbao-Sieyro, Yanira Florido, María Teresa Gómez-Casares

**Affiliations:** ^1^ Hematology Department, Hospital Universitario de Gran Canaria Dr. Negrin, Las Palmas de Gran Canaria, Spain; ^2^ Morfology Department, Universidad de Las Palmas de Gran Canaria, Las Palmas de Gran Canaria, Spain

**Keywords:** CALR, myeloproliferative neoplasms, type-1/2-like mutations

## Abstract

Identification of somatic frameshift mutations in exon 9 of the calreticulin gene (CALR) in myeloproliferative neoplasms (MPNs) in December of 2013 has been a remarkable finding. It has provided a new molecular diagnostic marker, particularly in essential thrombocythemia (ET) and primary myelofibrosis (PMF), where is the second most common altered gene after JAK2V617F. There are two main types of CALR mutants, type 1 and type 2, and there is evidence about their distinct clinical/prognostic implications, for instances, it is believed that favorable outcome might be restricted to type-1 in PMF. By using reasoned approaches, very recent publications have supported classifying the alternative mutants in type-1-like or type-2-like. If further studies confirm these results, new considerations may be taken into account in the molecular diagnosis of MPNs. This implies that precise mutation characterization must be performed and caution should be taken in screening technique selection. In this Editorial we summarize the current information regarding all this issues.

Identification of somatic frameshift mutations in exon 9 of *CALR* in myeloproliferative neoplasms (MPNs) has provided a new molecular diagnostic marker in essential thrombocythemia (ET) and primary myelofibrosis (PMF), where is the second most common alteration after *JAK2*^V617F^ [1]. There are two main types of *CALR* mutations and there is evidence about their distinct clinical/prognostic implications [2].

The most frequent mutations accounting for more than 80% of all, are type-1 variant, a 52 bp deletion (p.L367fs*46) and type-2, a 5bp TTGTC insertion (p.K385fs*47). Overall, type-1 is more frequent, but incidence of type-2 is higher in ET compared to PMF. Of the remaining, more than 50 different indels have been identified. First studies in MPNs showed that *CALR* compared to *JAK2* mutants were associated with lower hemoglobin level and leukocyte count, higher platelets and longer overall survival [1]. In ET, further publications demonstrated that patients carrying *CALR* mutations had lower risk of thrombosis than *JAK2/MPL*-mutated but there was no association with survival [3, 4]. However, *JAK2/CALR/MPL* mutational status in PMF is prognostically informative and outcome is better in CALR-mutated and worse in *JAK2*-positive or triple-negative patients [5, 6].

Recent evidence suggests that favorable outcome might be restricted to type-1 alteration in PMF [2], which may indicate the necessity of different therapeutic approaches. All frameshift mutations reported in the literature result in a +1bp frameshift. Type-1 mutation eliminates almost all negative charged aminoacids of the C-terminal domain whereas type-2 retains approximately half of them [1]. Bioinformatic analyses suggest additional functionally relevant structural differences between type-1 and type-2 mutants [7]. This could explain the mutant type distribution in PMF and ET and also the phenotypic and clinical differences. Mutations other than type-1/2 but resulting in 52bp deletions or 5bp insertions, have been reported. In some cases, they produce the same altered protein but an alternative in others. We, for instance, have identified a 52 bp deletion in ET (Figure 1) that is not the common type-1 since it starts at c.1090 (p.E364fs*49) [8]. It seems reasonable that mutations other that type-1/2 but with the same or similar protein effect should be included in each group in terms of studying clinical associations. Few recent publications have attempted classifying the alternative mutants in type-1-like or type-2-like. A statistical approximation algorithm was applied to obtain a structural protein prediction for each mutant [9]. They classified as type-2- like those mutants with higher α-helix content (more similar to wild-type), and as type-1-like those with significantly lower α-helix content. Survival was shorter in PMF patients with type-2/type-2-like vs type-1/type-1-like mutations, meaning that clinical/prognostic advantages of type-1 are extended to type-1-like. Other study categorized alternative mutants according to the grade of conservation of negatively charged aminoacids divided in three stretches [10]. Thus, type-2/type-2-like conserved the three stretches, type-1/type-1-like conserved one stretch, and other mutants conserved 2 stretches. Type-1-like mutations were mainly associated with PMF and significantly higher risk of myelofibrotic transformation in ET. Type-2-like was preferentially related to ET phenotype, low risk of thrombosis despite very-high platelet counts and indolent clinical course. Also, PMF carrying type-1-like had better survival compared to those with *JAK2*^V617F^.

**Figure 1:**
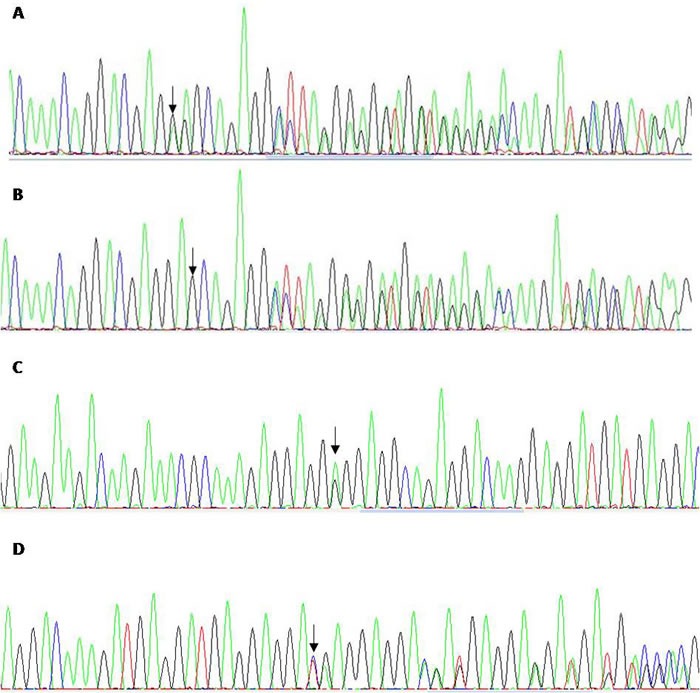
Sanger sequencing of CALR corresponding to a non classical 52 bp deletion c.1090_1141del from a patient with ET (**A**), the classical type 1 mutation from an ET case (**B**), a 9bp deletion c. 1177_1186del from oral mucosa of a patient with polyglobulia (**C**), an E380G variant in oral mucosa from a patient with transient thrombocytosis (**D**).

Hence, it seems that mutation characterization is critical to establish groups with specific clinical and prognostic features. There are several publications regarding methods for *CALR* mutation screening and some authors have suggested that fragment analysis determination may be sufficient for routine diagnostic purposes and even have developed real time PCR detection methods[11]. These screening techniques do not allow a precise characterization and sometimes it is not easy to determine the exact size of the insertion/deletion through fragment analysis. This is an important issue since we have found, as others, in-frame indel polymorphisms that could be misinterpreted as mutations if they are not properly characterized. We found a 9pb deletion at codon c.1177 of *CALR* in peripheral blood of a patient with polyglobulia and confirmed its germline nature by analyzing buccal swab DNA (Figure 1). We used high resolution melting as screening method because is a closed-tube technique, and its product can be directly used for subsequent sequencing [8]. Fragment analysis has the disadvantage that sequencing has to be performed from a new unlabeled PCR product, but mainly, it cannot discriminate point mutations. Although rare, nonsense mutations implying the loss of variable number of negatively charged amino acids of the C-terminus have been reported (i.e p.E374X, p.E380X, p.K391X) [12]. Besides, SNPs must be distinguished from mutations. We have also found in a patient with thrombocytosis, a nonsynonymous polymorphism, E380G (Figure 1), which has been previously characterized as a mutation [12]. However, we believe it may be most likely a polymorphism since it was a constitutional variant also detected in the oral mucosa and the thrombocytosis was transient.

According to recent studies, the C-terminus of type-1/2 mutants with its characteristic positive electrostatic charge has been shown to allow the association of mutant CALR with thrombopoietin receptor (c-MPL) and activate the JAK2 downstream pathway, whereas wild type and other mutants failed to interact with c-MPL [13, 14]. Therefore, clinical/biological implication of the point and nonsense variants remains to be determined as they may not contribute the same way as frameshift mutations.

On the other hand, either through fragment analysis or HRM as screening methods, we believe that sequencing is required since mutation characterization is essential to determine not only whether the alteration belongs to the clinically relevant types-1 or 2, or to the type-1/2-like but to distinguish polymorphisms from point/nonsense mutations that may be crucial for diagnosis.
